# Replicative senescence of mesenchymal stem cells causes DNA-methylation changes which correlate with repressive histone marks

**DOI:** 10.18632/aging.100391

**Published:** 2011-09-25

**Authors:** Anne Schellenberg, Qiong Lin, Herdit Schüler, Carmen M. Koch, Sylvia Joussen, Bernd Denecke, Gudrun Walenda, Norbert Pallua, Christoph V. Suschek, Martin Zenke, Wolfgang Wagner

**Affiliations:** ^1^ Helmholtz-Institute for Biomedical Engineering, Stem Cell Biology and Cellular Engineering, RWTH Aachen University Medical School, 52074 Aachen, Germany; ^2^ Institute for Biomedical Engineering - Cell Biology, RWTH Aachen University Medical School, 52074 Aachen, Germany; ^3^ Institute of Human Genetics, Medical Faculty, RWTH Aachen University, 52074 Aachen, Germany; ^4^ Interdisciplinary Centre for Clinical Research (IZKF) Aachen, RWTH Aachen University, 52074 Aachen, Germany; ^5^ Department of Plastic and Reconstructive Surgery, Hand Surgery, Burn Center, Medical Faculty, RWTH Aachen University, 52074 Aachen, Germany

**Keywords:** Mesenchymal stem cells, replicative senescence, long-term culture, colony forming units (CFU-f), karyotype, DNA-methylation, epigenetic

## Abstract

Cells in culture undergo replicative senescence. In this study, we analyzed functional, genetic and epigenetic sequels of long-term culture in human mesenchymal stem cells (MSC). Already within early passages the fibroblastoid colonyforming unit (CFU-f) frequency and the differentiation potential of MSC declined significantly. Relevant chromosomal aberrations were not detected by karyotyping and SNP-microarrays. Subsequently, we have compared DNA-methylation profiles with the Infinium HumanMethylation27 Bead Array and the profiles differed markedly in MSC derived from adipose tissue and bone marrow. Notably, all MSC revealed highly consistent senescence-associated modifications at specific CpG sites. These DNA-methylation changes correlated with histone marks of previously published data sets, such as trimethylation of H3K9, H3K27 and EZH2 targets. Taken together, culture expansion of MSC has profound functional implications - these are hardly reflected by genomic instability but they are associated with highly reproducible DNA-methylation changes which correlate with repressive histone marks. Therefore replicative senescence seems to be epigenetically controlled.

## INTRODUCTION

Fifty years ago, Leonhard Hayflick discovered that the number of cell divisions is limited for cells in culture [1] - after about 40 to 60 divisions the proliferation rate decays until the cells ultimately enter a senescent state. This phenomenon applies to all somatic cells in culture which are not immortalized.

It is often anticipated, that replicative senescence is caused by accumulation of cellular defects. Progressive shortening of telomeres or modified telomeric structures have been demonstrated to be an important trigger for replicative senescence [2]. It is however still controversially discussed if telomere shortening is really the initiating mechanism for replicative senescence or if it is rather a consequence of this process [3,4]. Alternatively, it has been suggested that replicative senescence is induced by oxidative stress, UV light or γ-irradiation [5,6]. This has led to the perception, that long-term culture leads to the accumulation of chromosomal aberrations resulting in cellular senescence, and this is of central interest for the emerging field of regenerative medicine [7].

Mesenchymal stem cells (MSC) comprise a subpopulation of multipotent adult stem cells that is able to differentiate into diverse mesodermal cell lineages [8]. Despite intensive research over the last decade there are still no reliable markers for this multipotent subset and therefore MSC are alternatively termed “mesenchymal stromal cells” [9]. The ease of culture expansion to large cell numbers has fuelled the hope for regenerative medicine. MSC are concurrently tested in a large number of clinical trials for a wide range of therapeutic applications (www.clinicaltrials.gov). So far, no critical side effects have been reported [10]. However, oncogenic transformation and transplant related tumor formation are like a “sword of Damokles” for cellular therapy [11]. Human MSC appear to be more resistant to *in vitro* transformation than murine MSC, with little genomic instability and no tumor-induction after transplantation [12-14]. Reports indicating malignant transformation of human MSC have recently been withdrawn as they were caused by cross-contamination with established immortalized cell lines [15,16]. However, MSC preparations may reveal transient aneuploidy without transformation [17] and highly proliferative culture conditions such as addition of human platelet lysate (HPL) might increase genomic instability upon culture expansion [18,19].

Long-term culture has also been suggested to induce epigenetic modifications [20-22]. CpG dinucleotides in the genomic DNA can be methylated at cytosine moieties. We have recently analyzed age-associated DNA methylation changes in MSC from human bone marrow [23] and dermal fibroblasts [24]using the HumanMethylation27 BeadChip microarray. This platform allows determination of DNA methylation levels at 27,578 unique CpG sites within more than 14,000 promoter regions. Overall, methylation patterns of MSC were maintained throughout both long-term culture and aging whereas highly significant modifications occur at specific CpG sites.

In continuation of this work we have now analyzed the molecular sequel of culture expansion of MSC from adipose tissue. We did not detect relevant chromosomal aberrations but there were significant differences in the DNA-methylation patterns of MSC derived from bone marrow and adipose tissue. Furthermore, senescence-associated DNA-methylation changes were observed in all samples and these correlated with repressive histone marks.

## RESULTS

### Long-term culture of MSC

**Figure 1:**
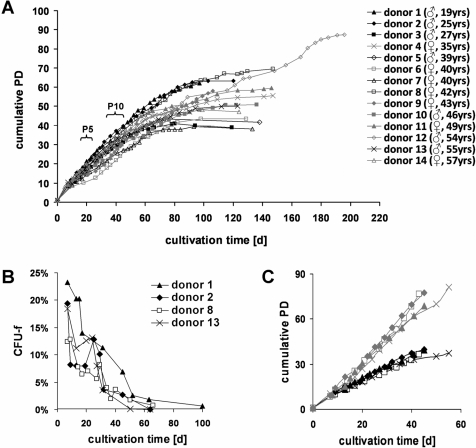
Long-term growth curves of MSC Mesenchymal stem cells were isolated from human adipose tissue of 14 donors and culture expanded until the cells reached a senescent state. Every cell passage is indicated by a point and the number of cumulative population doublings (PD) was calculated based on the ratio of cells seeded versus cells harvested per passage (**A**). In parallel, MSC were seeded in limiting dilution to determine the fibroblastoid colony forming unit (CFU-f) frequency for subsequent passages (**B**). To account for the fact, that the progeny of each passage is based on a decreasing percentage of highly proliferative cells, we have recalculated long-term growth curves on the basis of the number of CFU-f seeded versus cells harvested per passages (**C**; black: conventional PD; grey: CFU-f-adopted growth curves).

Mesenchymal stem cells from adipose tissue were expanded in culture medium with human platelet lysate (HPL) until they entered replicative senescence. Overall, the proliferation rate remained relatively high during the first two months before it declined. No age or gender-associated correlations were observed in long-term growth curves (figure 1A). All cell preparations entered growth arrest after 138 ±20 days and 53.8 ±14.4 cumulative population doublings (CPD). However, CPD do not necessarily correspond to the mean number of cell divisions as proliferation is heterogeneous in MSC: only a subset gives rise to new colonies upon passaging. To address the percentage of these highly proliferative cells we have tracked the frequency of fibroblastoid colony forming units (CFU-f) over subsequent passages. It was striking that the frequency of highly proliferative CFU-f declined continuously from about 20% in the first passage to less than 1% after two months (figure 1B). To accommodate the fact that the progeny of each passage is based on a decreasing number of highly proliferative cells, we have alternatively calculated the number of population doublings for each passage divided by the corresponding CFU-f frequency. This method of CFU-f-adjusted growth curves results in much higher numbers of cumulative population doublings (CPD^CFU-f^; figure 1C).

**Figure 2:**
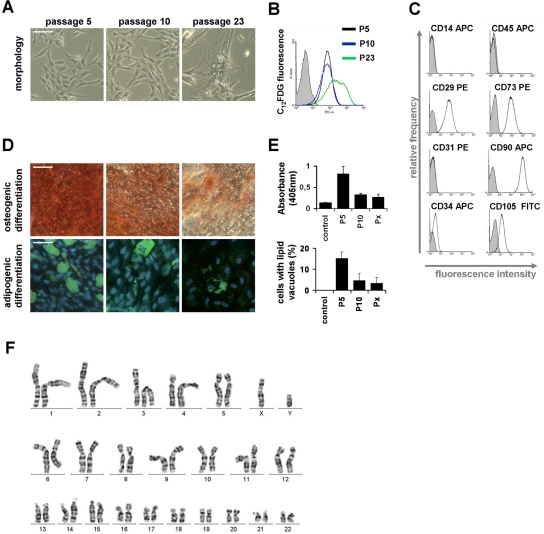
Changes of MSC during culture expansion Senescent MSC acquire a typical large and flat morphology, whereas no differences were observed at passage 5 and passage 10 (**A**). Expression of the senescence-associated β-galactosidase was detected with the fluorogenic substrate C_12_FDG and this biomarker for replicative senescence was positive in senescent passages but not in passage 5 and passage 10 (**B**). All MSC preparations displayed the typical immunophenotype (**C**) and could be induced towards osteogenic and adipogenic lineage (**D**). However, this *in vitro* differentiation potential decayed already between passage 5 and passage 10 (**E**; control is without induction medium; Px represents the corresponding senescent passage; all data are presented as mean ± SD; n = 4). A normal karyogram of MSC (after treatment with trypsin and Giemsa)is exemplarily presented (**F**). (scale bars = 100μm).

Senescent MSC displayed the typical large and flat cellular morphology. They also expressed senescence-associated (SA) beta galactosidase, which is a biomarker for cellular senescence and can be quantified with the fluorogenic substrate C_12_FDG. Neither morphological changes nor increase of SA-β-gal activity were observed during the exponential growth phase between passage 5 and passage 10 (figure 2A,B). The typical immunophenotypic pattern of MSC was maintained throughout culture expansion (CD14^−^, CD29^+^, CD31^−^, CD34^+/−^, CD45^−^, CD73^+^, CD90^+^ and CD105^+^; figure 2C). Another parameter for the definition of MSC is their *in vitro* differentiation potential [9]. Quantitative analysis of osteogenic and adipogenic differentiation revealed, that the differentiation potential decays already within early passages. Similar results have been described by many other groups before [25-28] (figure 2D,E).

### Analysis of genetic aberrations

Genomic stability is an important concern for clinical use of MSC [7,17]. Therefore, we have compared the karyotypes of four MSC preparations at early passage and passage 10. We have also included passage 8 and passage 32 of donor 12 as these cells entered the senescent state later than the other cell preparations (figure 2F; table 1; in total 167 mitoses were analyzed). All MSC revealed a normal 2n karyotype with no consistent chromosomal aberrations. Only 3 mitoses in two samples of early passage showed additional chromosomes and these might represent artifacts.

**Table 1:** Karyotypic analysis

sample	passage	days in culture (no. of CPD)	no. of mitoses (no. of slides)	karyotype	abnormalities
donor 4	5	25d (22.7)	20 (15)	46,XX	1 x 47,XX,+C 1x 47,XX,+mar
	10	46d (34.8)	16 (7)	46,XX	-
donor 8	5	21d (18.8)	11 (4)	46,XX	1x 47,XX,+19
	10	40d (32.8)	22 (10)	46,XX	-
donor 10	3	14d (14.2)	11 (4)	46,XY	-
	10	44d (30.0)	22 (10)	46,XY	-
donor 11	5	25d (22.9)	14 (6)	46,XX	-
	10	46d (36.2)	14 (6)	46,XX	-
donor 12	8	36d (28.6)	15 (6)	46,XY	-
	32	185d (85.9)	22 (10)	46,XY	-
+C = with additional C-group chromosome (medium-sized, submetacentric human chromosomes); +mar = with marker chromosome (structurally abnormal chromosome in which no part can be identified)					

To detect smaller chromosomal lesions we used single nucleotide polymorphism (SNP) arrays (table 2). This method does not account for the heterogeneity within cells in culture, but it can detect small chromosomal aberrations which might result in growth advantage of individual subclones. Some regions showed gains and losses at 200 kb resolution - most of them were consistent between early and later passage and may therefore be donor specific. Few aberrations were detected only in either early or late passage and these copy number variations were restricted to small genomic regions. These few gains and losses are most likely due to technical noise or to differences in the reference DNA pool. Taken together, MSC from adipose tissue are relatively resistant to genomic instabilities even under highly proliferative growth conditions with HPL. This corresponds to our results for MSC from bone marrow [14] and those of many other groups [12,13,17,29,30].

**Table 2:**
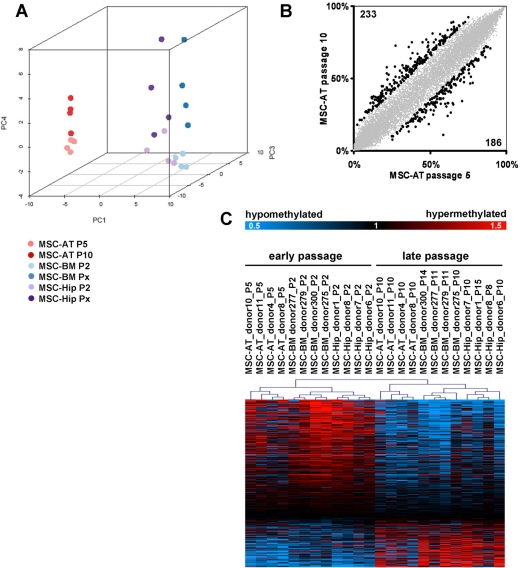


### DNA methylation profiling

**Figure 3:**
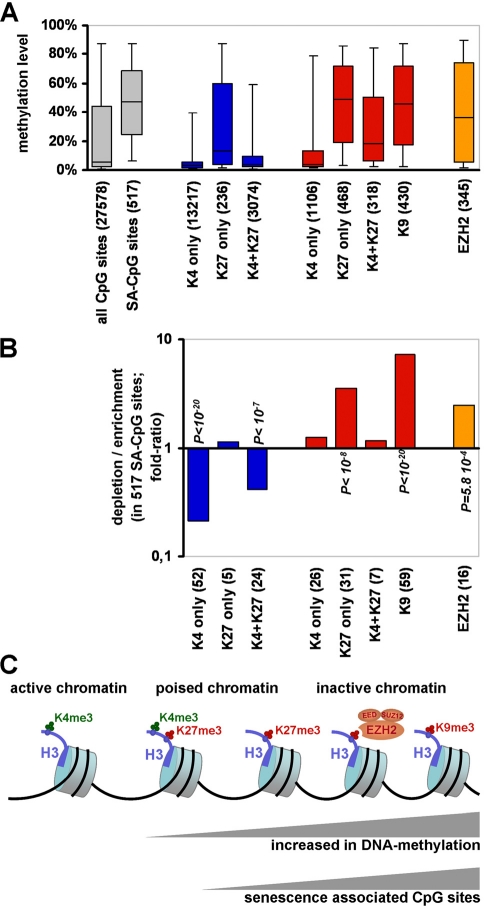
Senescence-associated modifications in the DNA-methylation pattern DNA-methylation profiles were analyzed with the HumanMethylation27 BeadChip microarray which represents 27,578 unique CpG sites. MSC derived from adipose tissue (MSC-AT) were compared with those derived from bone marrow, which was either aspirated from the iliac crest (MSC-BM) or taken from the caput femuris upon hip replacement (MSC-Hip). Unsupervised principal component analysis (PCA) clearly separated DNA-methylation profiles according to the tissue of origin in the first dimension (PC1), whereas the forth component (PC4) discerned early and late passage (**A**). Scatterplot comparison of passage 5 and passage 10 in MSC-AT revealed that 233 CpG sites are more than 15% hyper-methylated whereas 186 CpG sites are more than 15% hypo-methylated at passage 10 (**B**). Significance Analysis of Microarray (SAM) was used to select 517 senescence-associated CpG sites (FDR = 4.8%) and these are presented as a heatmap (**C**; data were divided by the mean of each row for graphical presentation).

Subsequently, we analyzed if MSC acquire epigenetic modifications upon expansion. DNA methylation profiles of early passage (P5) and later passage (P10) of the four samples were compared using the Human-Methylation27 BeadChip microarray. We have previously described senescence-associated DNA- methylation changes in MSC from bone marrow which were either isolated from bone marrow aspirates (MSC-BM) or from the caput femoris upon hip fracture of elderly donors (MSC-Hip) [23]. Raw beta-values of adipose tissue and bone marrow derived datasets were quantile normalized together for subsequent analysis of epigenetic differences. Unsupervised principal component analysis (PCA) clearly separated DNA-methylation profiles according to the tissue of origin in the first dimension indicating that there are marked differences in their epigenetic make up. The second axis separated the samples predominantly according to gender (supplemental figure 1). Notably, the forth component (PC4) separated the DNA-methylation profiles of MSC in those of early and later passages (figure 3A) and this demonstrates that expansion of MSC has a very consistent impact on DNA-methylation profiles.

To further elaborate epigenetic differences between MSC from different tissues we have only considered data of early passages for a direct comparison of MSC-AT *versus* MSC-BM and MSC-Hip. Scatter plot analysis revealed that 1021 CpG sites were more than 15% hyper-methylated whereas 1175 CpG sites were more than 15% hypo-methylated in MSC-AT (supplemental figure 2). Alternatively we have used the Bayesian method Significance Analysis of Microarray (SAM) to estimate the false discovery rate (FDR): 8,370 of the 27,578 CpG sites represented on the microarray were differentially methylated (FDR = 5%). We have focused on the 539 most significantly differentially methylated CpG sites (FDR < 0,0001%) for functional categorization of genes which correspond to these genomic sites. Genes with tissue-specific DNA-methylation changes were most significantly enriched in the Gene Ontology categories for nutrient level, lipid modification and glucose metabolism (supplemental figure 3A). This may reflect the different function of these tissues. The results demonstrate that MSC from different tissues have considerable different DNA-methylation profiles although their growth pattern, immunophenotype and *in vitro* differentiation potential are quite similar.

Subsequently, we have focused on long term culture-associated changes in MSC-AT. Overall the DNA-methylation level remained relatively constant in comparison of passage 5 and passage 10, whereas 233 CpG sites became more than 15% hyper-methylated and 186 CpG sites were hypo-methylated upon replicative senescence (figure 3B). For further analysis, we have focused on those CpG sites with the most significant senescence-associated changes in all MSC preparations. 517 CpG sites were consistently differentially methylated in early *versus* late passages in MSC-AT, MSC-BM and MSC-Hip (pair wise SAM; FDR = 4.8%; 156 CpG sites hyper-methylated and 361 CpG sites hypo-methylated upon replicative senescence; figure 3C). Genes associated with CpGs that were significantly differentially methylated upon replicative senescence included distal-less homeobox 5 (*DLX5*), cyclin-dependent kinase inhibitor 2B (*CDKN2B*) and homeobox D10 (*HOXD10*). Gene Ontology analysis revealed that senescence-associated DNA-methylation changes were significantly enriched in genes for defense response and epidermal development (supplemental figure 3B). This accumulation of epigenetic modifications in developmental genes supports the notion that replicative senescence is a developmental process.

### Senescence-associated DNA-methylation changes correlate with repressive histone marks

**Figure 4:**
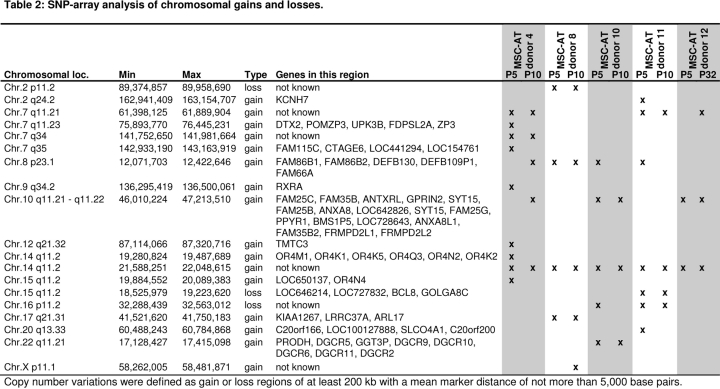
Senescence-associated DNA-methylation changes correlate with repressive histone marks DNA-methylation profiles of MSC were compared with datasets for H3K4me3 and H3K27me3 in human ESC [34] (indicated in blue); H3K4me3, H3K9me3 and H3K27me3 in human MSC-AT [35] (red) and targets of the Enhancer of Zeste Homolog 2 (EZH2) in human MSC-BM [36] (orange). The overall DNA-methylation level of all 27,578 CpG sites on the microarray was much lower in comparison to the 517 senescence-associated (SA) CpG sites and to those which have been assigned for H3K27me3, H3K9me3 and targets of EZH2 (**A**; Box plots represent the 25^th^ and 75^th^ percentile for each subset and whiskers show the 5% and 95% percentiles). Notably, the 517 senescence-associated DNA-methylation changes were significantly enriched in regions with H3K27me3, H3K9me3 and EZH2 targets (**B**). The graphic illustrates that repressive histone marks are associated with higher levels of DNA-methylation and that senescence-associated modifications are enriched in these regions (**C**).

The DNA-methylation pattern has been shown to be linked to histone modifications - especially methylation of histone H3 [31-33]. Therefore, we have compared our DNA-methylation profiles of MSC-AT, MSC-BM and MSC-Hip with previously published data on trimethylated H3K4 (H3K4me3) and H3K27me3 in human embryonic stem cells (hESC) [34], H3K4me3, H3K9me3 and H3K27me3 in human MSC-AT [35] and targets of the Enhancer of Zeste Homolog 2 (EZH2) in human MSC-BM [36]. Initially, we have compared the overall methylation level in all 27,578 CpG sites on the microarray with the methylation levels of those CpG sites which have been assigned for histone modifications. The median methylation level of all CpG sites was 5.4%. Those CpG sites that have previously been associated with the H3K4me3 mark in hESC and MSC-AT showed a significantly lower methylation level (2.9% and 4.1% respectively). In contrast, CpG sites which carried only H3K27me3 (13.6% and 49.2%) or H3K9me3 (45.8%) were associated with much higher levels of methylation. EZH2 mediates H3K27 trimethylation and accordingly, EZH2 targets were also linked to significantly higher DNA-methylation levels (36.1%) (figure 4A). These results are in line with the notion, that DNA-methylation is associated with absence of H3K4me3 and the presence of H3K9me3 [31,32,37,38].

Subsequently, we have analyzed if the 517 senescence-associated DNA-methylation changes are enriched in regions with these specific histone modifications. Statistical analysis with hypergeometric distribution revealed that they are significantly enriched with H3K27me3 (P = 1.2 * 10^−9^), H3K9m3 (P < 10^−20^) and EZH2 target regions (P = 5.8 * 10^−^) (figure 4B). This is in line with the fact that the senescence-associated CpG sites displayed a much higher median methylation level (47.2%) compared to all CpG sites on the microarray. Thus, senescence-associated DNA-methylation changes are associated with repressive histone marks and with targets of the polycomb protein EZH2. These results indicate that epigenetic histone modifications contribute to senescence-associated changes in the DNA-methylation pattern or *vice versa* (figure 4C).

## DISCUSSION

Human MSC are relatively stable towards chromosomal aberrations whereas long-term culture induces highly reproducible epigenetic modifications at specific sites in the genome. These epigenetic modifications may govern senescence-associated functional changes such as loss of differentiation potential upon several passages. Therefore, culture expansion and passaging clearly have to be taken into account for cellular therapy.

Replicative senescence does not occur synchronously in cell preparations. We have demonstrated that the percentage of colony initiating cells declines already within the first passages. This indicates that many cells have a restricted proliferative capacity in the exponential growth phase but these are quickly outgrown by more proliferative subpopulations. Therefore typical morphological changes or senescence-associated β-galactosidase are hardly observed until these proliferative subpopulations reach senescence, too. So far, calculation of cumulative population doublings is based on the ratio of cells seeded and cells harvested at each passage - this however does not consider heterogeneity of replicative senescence. In fact the progeny of MSC is put forth by a subset of cells, which consequently undergo more cell divisions than expected. On the other hand our CFU-f adopted growth curves are solely based on highly proliferative cells and the real average number of cell divisions lies between the conventional and CFU-f adopted long-term growth curves. This exemplifies that it is not an easy task to track replicative senescence of MSC *in vitro*[11].

Apart from this continuous loss of proliferative subpopulations, it is of central importance if specific clones confer selective growth advantage or promote cell transformation [17]. Addition of 10% human platelet lysate results in very fast proliferation of MSC-AT and this may further trigger accumulation of genomic aberrations [18]. Our cell preparations revealed a normal karyotype although abnormalities were observed in few individual cells and SNP-arrays did hardly detect gains or losses of certain subpopulations. Since all MSC preparations reached a senescent state upon long-term culture and stopped proliferation, the detected genetic alterations did not promote cell transformation but rather confer to growth disadvantages due to DNA damage. These observations are in line with several other studies, which indicated that human MSC are relatively resistant to genomic aberrations and transformation in culture expansion [12-14,17,30]. Despite absence of major clinical side effects a potential risk remains that transplantation of MSC may harbor some tumorigenic potential - especially in autologous transplant settings. On the other hand, it is highly questionable if karyotypic analysis or SNP-arrays can safeguard this issue [17].

MSC are characterized by plastic adherent growth, a panel of immunophenotypic surface markers and their ability to differentiate towards osteogenic, adipogenic and chondrogenic lineage [9]. All our MSC preparations passed these criteria, but this relative superficial definition of MSC does not account for the differences which arise from different methods for cell preparations [39]. We and other authors have demonstrated, that MSC from adipose tissue and bone marrow have different gene expression profiles [40-42]. Here, we have shown that the DNA-methylation profile differs also markedly between MSC from adipose tissue and bone marrow. For fibroblasts, it has even been shown that DNA methylation profiles of cells from the same dermal region clustered closely together indicating that fibroblasts maintain positional memory despite *in vitro* culture [24]. Hence, the tissue of origin appears to be imprinted in cell preparations and this might relate to functional differences between MSC preparations.

In this study, we have demonstrated that specific changes in the DNA-methylation pattern arise already during the exponential phase of culture expansion (between passage 5 and passage 10). Many of these senescence-associated changes were related to those between early and senescent passage in MSC from bone marrow [23]. This highly consistent modification at specific CpG sites - many of which are associated with developmental genes - indicates that replicative senescence represents a developmental process, rather than a random accumulation of cellular defects.

Long-term repression in the course of replicative senescence requires the ability to maintain localized silencing through many cell divisions. Therefore, DNA-methylation provides an ideal mechanism as the methylation pattern is established on the newly synthesized DNA strand by DNA methyltransferase 1 (DNMT1) [43,44]. On the other hand, it has been shown that DNA-methylation patterns are better correlated with histone methylation patterns than with the underlying genome sequence context [31,32,37,38]: DNA-methylation is associated with absence of H3K4me3 and presence of H3K9me3. This association was also clearly observed by combination of our DNA-methylation data and previous published ChIP-seq and ChIP-chip datasets in MSC-AT and MSC-BM. More importantly, senescence-associated DNA-methylation changes were highly significantly enriched in regions with H3K9me3, H3K27me3 and EZH2 targets. In contrast, H3K4me3 has been suggested to disrupt the contacts between nucleosomes making DNA sequences accessible for zinc finger proteins which protect CpG islands from DNA methylation [38]. The histone methyltransferase EZH2, a component of the polycomb-repressive complex 2 (PRC2) has previously been implicated in replicative senescence [45,46,46,47]: EZH2 levels are down-regulated in senescent cells [48]. Targeting of EZH2 with short hairpin interference results in senescence whereas over-expression bypasses senescence [49]. On the other hand, EZH2 overexpression is associated with a variety of cancers and inhibition of polycomb group proteins has been suggested as a potential therapeutic strategy [50-52]. Components of PRC1 are also capable of delaying the onset of senescence in fibroblasts [48,53] and the precise composition of senescence-regulating complexes is yet unknown. Furthermore, regulatory mechanisms gain another dimension of complexity by various microRNAs that have been implicated in regulation of replicative senescence [27,54-56].

If replicative senescence represents an epigenetically regulated developmental program then the question remains how this confers a selective advantage. It might resemble a protective mechanism against accumulation of cellular defects which might ultimately result in cell transformation and tumor formation [57]. Since the discovery of the “Hayflick limit” it has also been speculated if it is related to the aging process of the organism [58-61]. We have previously demonstrated a moderate but significant concordance in molecular changes upon long-term culture *in vitro* and aging *in vivo* - this was observed on gene expression level [62] and DNA-methylation level [23,24]. It has been shown that polycomb group protein targets (PCGT) are far more likely to become methylated with age[63]. We did not observe a significant association of senescence-associated DNA-methylation changes with the 69 age-associated CpG sites described in this study (results not shown) but we have demonstrated that they are associated with targets of EZH2 which represents a component of the PCR2. Other authors have shown that age-associated hyper-methylation occurs predominantly at bivalent chromatin domain promoters in CD4^+^ T-cells and CD14^+^ monocytes [64]. Our study demonstrates that senescence-associated DNA-methylation changes in MSC are rather associated with repressive histone marks than with bivalent modifications (H3K4me3 and H3K27me3). Epigenetic modifications upon replicative senescence *in vitro* and aging *in vivo* seem to be related but it might be expected that the complex regulatory mechanisms differ in these two processes [7,65].

In summary, expansion of MSC is associated with a systematic modulation of the epigenetic chromatin structure at specific sites in the genome but the precise regulation of this process is yet unknown. We have demonstrated that senescence-associated hyper-methylation and hypo-methylation were often observed in regions with H3K9me3, H3K27me3 and targets of EZH2. Therefore it is conceivable, that replicative senescence is regulated by protein complexes such as PRC2. Notably, replicative senescence appears to be reversible: induced pluripotent stem cells as well as ESC and germline cells do not show signs of replicative senescence in long-term culture [66]. Furthermore, replicative senescence can be pharmacologically decelerated: cells retain their proliferative capacity during cell cycle arrest in the presence of rapamycin, an inhibitor of mTOR [67,68]. Selective manipulation of this process might ultimately facilitate expansion of more potent cell populations for regenerative medicine.

## METHODS

### Isolation of MSC

MSC from adipose tissue (MSC-AT) were isolated from lipoaspirates at RWTH Aachen University after written consent of all participants. These studies were performed according to the guidelines of the Ethics Committee of the RWTH Aachen University Medical School, Germany (Permit number 163/07) that specifically approved this study. Lipoaspirates were washed in 9 g/L NaCl, digested with 2 g/L collagenase type I (PAA, Pasching, Austria) and passed through a 100 μm cell strainer as described before [19]. Culture medium consisted of Dulbecco?s Modified Eagles Medium-Low Glucose (DMEM-LG; PAA) with 2 mM L-glutamine (Sigma Aldrich, St. Louis, MO, USA), 100 U/mL penicillin/streptomycin (pen/strep; Lonza, Basel, Switzerland) and 10% human platelet lysate (HPL) which was pooled from five platelet units of healthy donors as described before [69]. Cells were cultured at 37°C in a humidified atmosphere containing 5% carbon dioxide with medium changes twice per week. Freshly isolated cells were seeded in parallel in limiting dilutions in two 96-well plates (100, 300, 1,000 and 3,000 cells per well; 48 replica for each condition) to determine the initial fibroblastoid colony-forming unit (CFU-f) frequency.

### Long-term culture of MSC

MSC were always harvested by trypsinisation upon 80% confluent growth, counted with a Neubauer counting chamber (Brand, Wertheim, Germany) and additionally with a CASY cell counter (Schärfe System, Reutlingen, Germany) and re-seeded in a density of 10,000 cells/cm^2^ in culture flasks (Nunc Thermo Fisher Scientific, Langenselbold, Germany). Cell Population doublings per passage (PDP) and cumulative population doublings (CPD) were calculated from the first passage onward by the following formula [19]: PDP*_i_* = log_2_ (h*_i_/*s*_i_*); CPD = sum_i = 1…n_ PDP*_i_*, where n is the total number of passages; s*_i_* is the number of cells seeded at passage *i* and h*_i_* is the number of cells harvested in passage *i*. In parallel, cells were seeded in limiting dilutions in96-well plates (1, 3, 10 and 30 cells per well; 48 replica for eachcondition) to calculate the CFU-f frequency over subsequent passages. After 14 days all wells were scored for more than 50% confluent growth. Based on these results, we alternatively calculated CFU-f corrected doublings per passage: PDP^CFU-f^ = (h*_i_ /*(s_i_*CFU-f*_i_*) and CPD^CFU-f^ = sum_i = 1…n_ PDP^CFU-f^*_i_*.

### Senescence associated β-galactosidase staining

Expression of pH dependent senescence associated β-galactosidase (SA-β-gal) activity was analyzed with a fluorescence-based method for quantitative and sensitive analysis by flow-cytometry as described before [70]. In brief, Bafilomycin A1 (Sigma, St Louis, MO, USA)prevents lysosomal acidification and subsequently 5-dodecanoylaminofluorescein di-β-D-galactopyrano?side (C_12_FDG, Invitrogen, Eugene, OR, USA) was used as fluorogenic substrate for β-galactosidase.

### Immunophenotypic analysis

Surface marker expression was analyzed on a FACS canto II (Becton Dickinson [BD] , San Jose, USA) upon staining with the following antibodies as described before [19]: CD14-allophycocyanin (APC, clone M5E2, BD), CD29-phycoerythrin (PE, clone MAR4, BD), CD31-PE (clone WM59, BD), CD34-APC (clone 8G12, BD), CD45-APC (clone HI30, BD), CD73-PE (clone AD2, BD), CD90-APC (clone 5E10, BD), CD105-fluorescein isothiocyanate (FITC, clone MEM-226 Immuno Tools).

### *In vitro* differentiation

Osteogenic, adipogenic and chondrogenic differentiation of MSC were simultaneously performed as described before [8,27]. After three weeks, osteogenic differentiation was analyzed by Alizarin Red staining and quantified with a Tecan infinite M200 plate-reader (405 nm) [71]. Adipogenic differentiation was analyzed by staining of fat droplets with the green fluorescent dye BODIPY (4,4-difluoro-1,2,5,7,8-pentamethyl-4-bora-3a,4a-diaza-s-indacene) counter-stained with DAPI (4',6-Diamidin-2-phenylindol; both Molecular Probes, Eugene, Oregon, USA). Fluorescence microscopic pictures were always taken from five randomly chosen areas and the percentage of cells with fat droplets was determined as described before [19,69]. Images were captured at room temperature using a Leica DM IL LED microscope (Leica, Wetzlar, Germany) with a 10x dry objective (numerical aperture: 0.3; Leica) and a camera (Leica DFC420C) equipped with Leica application suite 3.3.1 software (Leica).

### Cytogenetic analysis

To investigate structural and numerical chromosomal alterations we have performed conventional karyotyping of cultured MSC using GTG banding at 300 to 400 band level. Metaphase spreads were prepared using standard procedures of hypotonic treatment and methanol/acetic acid fixation (3:1). GTG -banding comprising a trypsin pretreatment was carried out according to standard protocols.Microscopy was performed with Axioplan fluorescence microscope (Carl Zeiss, Jena, Germany) and IKARUS™ and ISIS™ digital imaging systems (MetaSystems, Altlussheim, Germany). 11 to 22 GTG banded metaphases were analyzed for each sample.

### SNP array analysis

Copy number changes of DNA fragments were analyzed with the Affymetrix Genome-Wide human single nucleotide polymorphism (SNP) array 6.0 which contains more than 1.8 million genetic markers. Genomic DNA was isolated from 10^6^ cells using the DNA Blood Midi-Kit (Qiagen, Hilden, Germany). DNA quality was assessed with a NanoDrop ND-1000 spectrometer (NanoDrop Technologies, Wilmigton, Del) and agarose gel electrophoresis. For a reference, we have used a DNA pool of leucocytes of 100 donors. 250 ng of genomic DNA were amplified, labelled and hybridized to the SNP Array according to the manufacturer's instructions (Affymetrix Inc., Santa Clara, CA, USA). The arrays were stained and washed using a fluidics Station 450 and scanned with a GeneChip Scanner 3000 7G (both Affymetrix). GeneChip® Operating Software (GCOS), Genotyping Console v3.0.1 and Chromosome Analysis Suite (ChAS) software (all Affymetrix) were used for data analysis. Copy number variations were defined as gain or loss regions of at least 200 kb with a mean marker distance of not more than 5,000 base pairs.

### DNA-methylation profiling

NA methylation profiles were analyzed using the HumanMethylation27 BeadChip (Illumina, San Diego, USA) as described before [23]. In brief, about 200 ng of bisulfite converted DNA were applied per BeadChip according to the manufacturer's instructions. After single-base extension using DNP- and Biotin-labeled ddNTPs, the array was fluorescently stained, scanned, and the intensities of non-methylated and methylated bead types measured. Initial data analysis was performed with the BeadStudio Methylation Module. DNA methylation values, described as beta values, are recorded for each locus in each sample and the complete dataset has been deposited in NCBIs Gene Expression Omnibus (GEO, http://www.ncbi.nlm.nih.gov/geo/) and are accessible through GEO Series accession numbers GSE26519.

### Bioinformatic analysis of DNA methylation data

DNA-methylation profiles of MSC-AT were analyzed in conjunction with the previously published data on MSC from bone marrow (GSE17448). The combined dataset was quantile normalized to minimize chip effects [72]. Principalcomponents analysis (PCA) wascalculated with prcomp in R package stats. Hierarchical clustering by Euclidian distance was performed using the MultiExperiment Viewer (MeV, TM4) [73]. Initially, we have selected CpG sites with differences in average beta value greater than 0.15 (more than 15% difference in mean methylation level) as this method is relatively resistant to potential confounding BeadChip effects. To select significantly differentially methylated CpG sites we used the Significance Analysis of Microarray (SAM) method [74] at a false discovery rate of less than 5%. Genes associated with the differentially methylated CpG sites were classified by Gene Ontology analysis using GoMiner software (http://discover.nci.nih.gov/gominer/).

DNA-methylation profiles were subsequently compared with published chromatin immunprecipitation (ChIP) results: H3K4me3 and H3K27me3 were analyzed in human embryonic stem cells (hESC; sequenced with Illumina technology) [34], H3K4me3, H3K9me3 and H3K27me3 were analyzed in human MSC-AT (hybridized to NimbleGen RefSeq Promotor Array) [35] and EZH2 targets were analyzed in human MSC-BM (hybridized to NimbleGen RefSeq promoter array) [36]. For comparison, corresponding CpG sites on the HumanMethylation27 BeadChip were matched by RefSeq ID and subsequently by mapping to the chromosomal position (within 100 base pairs up- and down-stream of the target sequence).

### Statistics

Quantification of *in vitro* differentiation assays is presented as mean ± standard deviation (SD). Probability of enrichment/depletion of senescence-associated DNA-methylation changes was estimated by hypergeometric distribution. Representation of differentially methylated genes in Gene Ontology categories was analyzed by Fischer?s Exact p-value.

## SUPPLEMENTAL FIGURES

Supplemental Figure 1 Principal component analysis of DNA-methylation profiles Principal component 1 (PC1) separates the cell preparations predominantly according to the tissue of origin whereas PC2 discerns male and female samples.

Supplemental Figure 2 Comparison of MSC from adipose tissue and bone marrow Scatterplot comparison of early passages of MSC-AT versus those from MSC-BM and MSC-Hip (**A**). Heat map presentation of DNA-methylation at the 539 most significantly differentially regulated CpG sites between MSC from adipose tissue and bone marrow (**B**; FDR = 0).

Supplemental Figure 3 Gene Ontology categorization of differentially methylated genes Genes associated with 539 differentially methylated CpG sites between MSC from adipose tissue and bone marrow (**A**) or with 517 SA-CpG sites (**B**) were categorized by Gene Ontology and the most significantly over-represented categories are depicted.
