# Supplementary figures and images for: Replicative senescence of mesenchymal stem cells causes DNA-methylation changes which correlate with repressive histone marks
Source: Aging (Albany NY). 2011 Sep 25;3(9):873–88. doi: 10.18632/aging.100391 (PMC3227452; doi:10.18632/aging.100391)

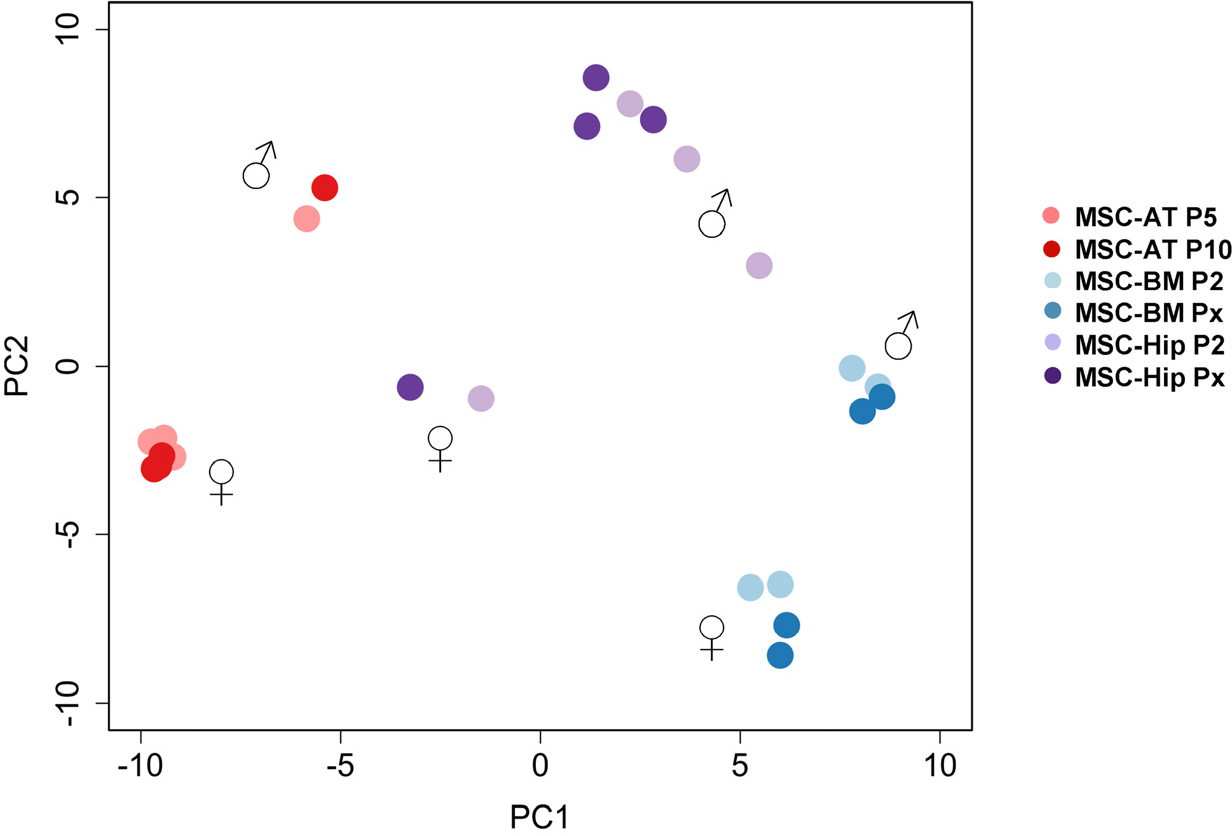

Supplement: Supplemental Figure 1 — Principal component 1 (PC1) separates the cell preparations predominantly according to the tissue of origin whereas PC2 discerns male and female samples. [file aging-03-873-s001.tif]

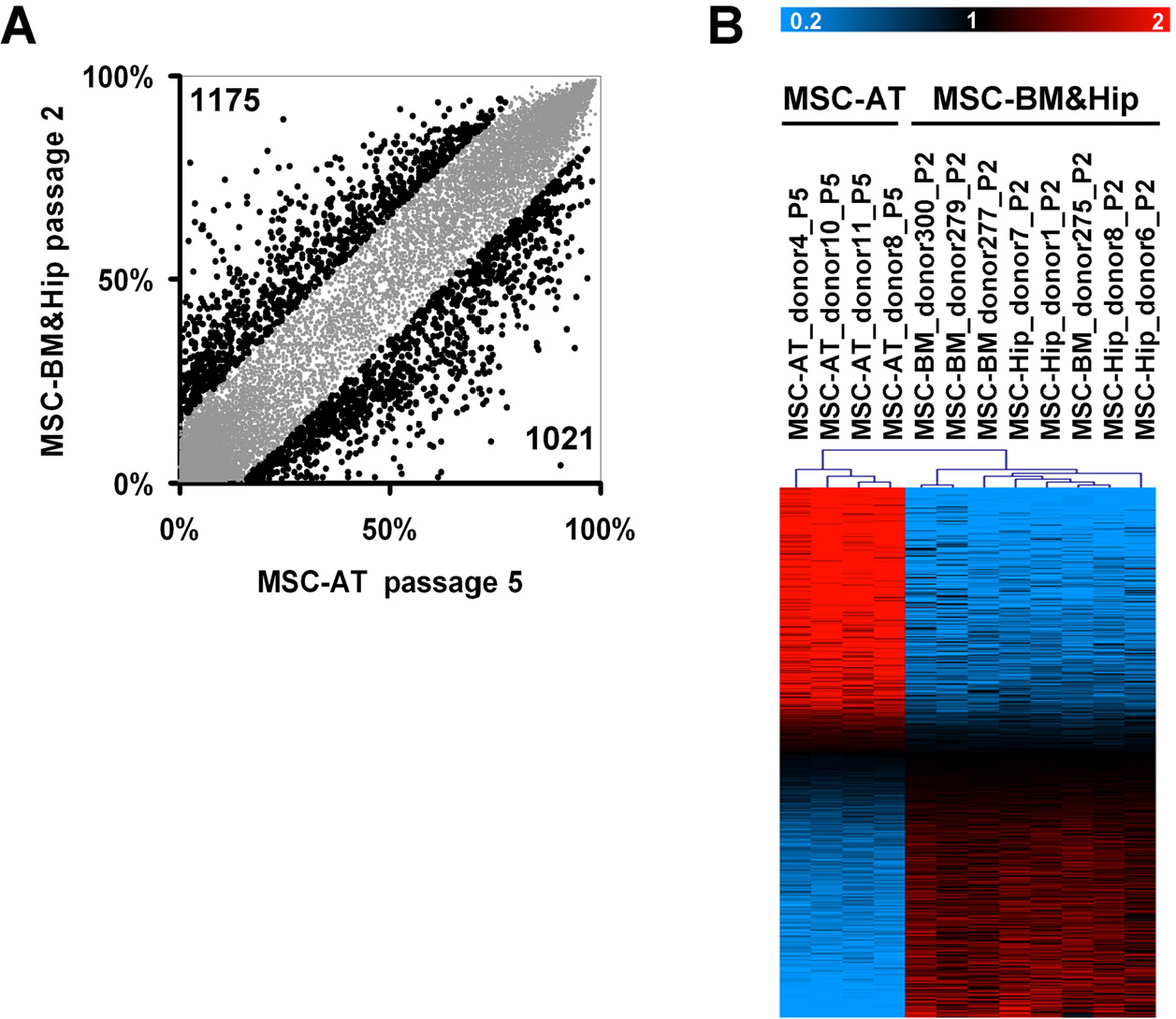

Supplement: Supplemental Figure 2 — Scatterplot comparison of early passages of MSC-AT versus those from MSC-BM and MSC-Hip (A). Heat map presentation of DNA-methylation at the 539 most significantly differentially regulated CpG sites between MSC from adipose tissue and bone marrow (B; FDR = 0). [file aging-03-873-s002.tif]

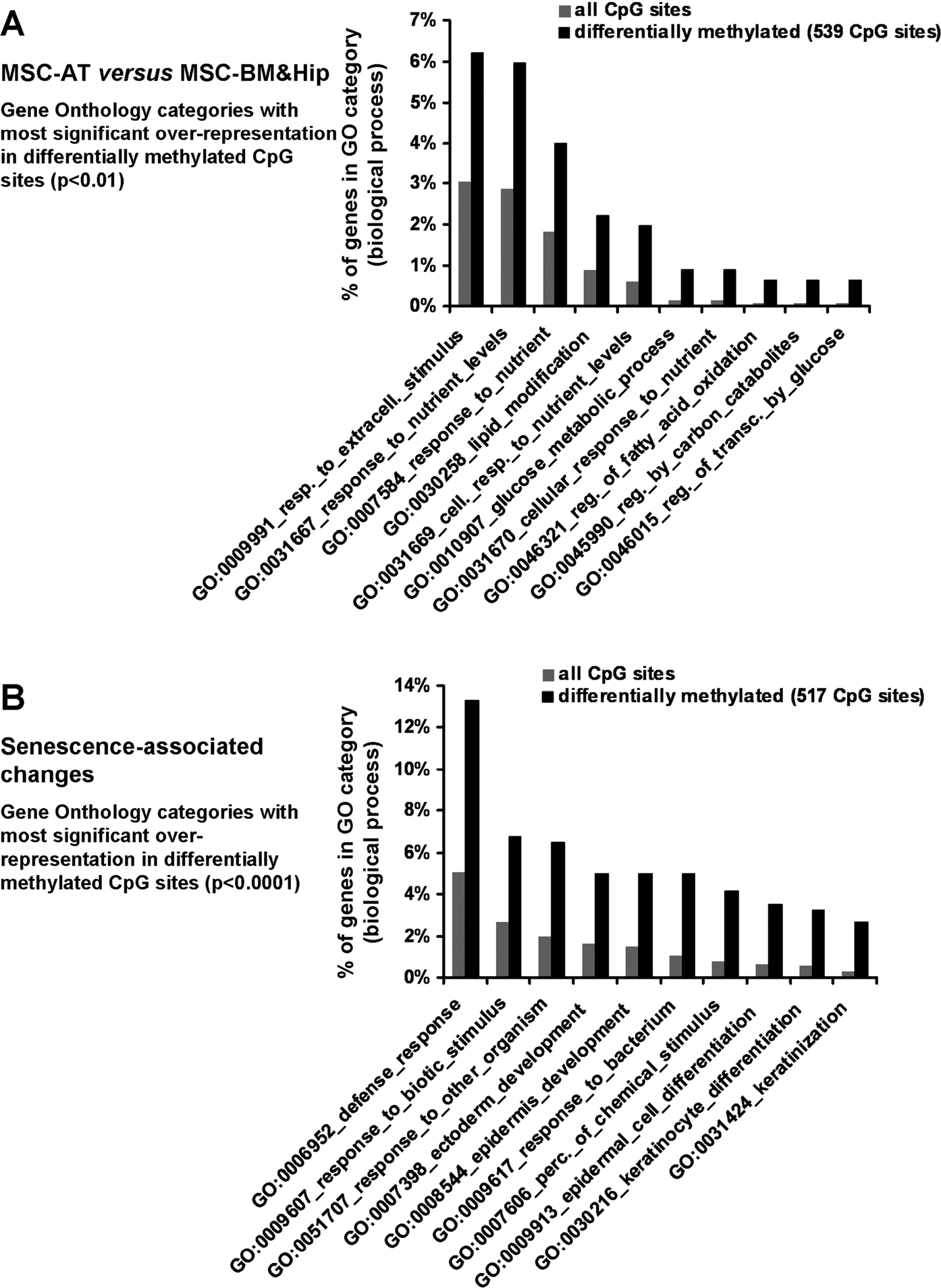

Supplement: Supplemental Figure 3 — Genes associated with 539 differentially methylated CpG sites between MSC from adipose tissue and bone marrow (A) or with 517 SA-CpG sites (B) were categorized by Gene Ontology and the most significantly over-represented categories are depicted. [file aging-03-873-s003.tif]
